# Disparities in Height and Urban Social Stratification in the First Half of the 20th Century in Madrid (Spain)

**DOI:** 10.3390/ijerph16112048

**Published:** 2019-06-10

**Authors:** Carlos Varea, Elena Sánchez-García, Barry Bogin, Luis Ríos, Bustar Gómez-Salinas, Alejandro López-Canorea, José Miguel Martínez-Carrión

**Affiliations:** 1 Department of Biology, Faculty of Sciences, Madrid Autonomous University, 28049 Madrid, Spain; elena.sanchezg01@estudiante.uam.es (E.S.-G); bustar.gomez@estudiante.uam.es (B.G.-S); alejandro.lopezcanorea@estudiante.uam.es (A.L.-C.); 2 School of Sport, Exercise & Health Sciences, Loughborough University, Loughborough LE11 3TU, UK; B.A.Bogin@lboro.ac.uk; 3 Department of Physical Anthropology, Aranzadi Zientzia Elkartea, 20014 Donostia, Gipuzkoa, Spain; mertibea@yahoo.com; 4 Department of Applied Economics, Faculty of Economics and Business, Murcia University, 30100 Murcia, Spain; jcarrion@um.es

**Keywords:** life cycle, plasticity, anthropometry, secular trends, inequality, community effect

## Abstract

Adult height is the most commonly used biological indicator to evaluate material and emotional conditions in which people grew up, allowing the analysis of secular trends associated with socio-economic change as well as of social inequalities among human populations. There is a lack of studies on both aspects regarding urban populations. Our study evaluates the secular trends and the disparities in height of conscripts born between 1915 and 1953 and called-up at the age of 21 between 1936 and 1969, living in districts with low versus middle and high socio-economic conditions, in the city of Madrid, Spain. We test the hypothesis that urban spatial segregation and social stratification was associated with significant differences in height. Results show that height increased significantly during the analysed period, both among conscripts living in the middle- and upper-class districts (5.85 cm) and in the lower-class districts (6.75 cm). The positive secular trend in height among conscripts from middle- and upper-class districts was sustained throughout the period, but the trend in height among the lower class fluctuated according to social, political, and economic events. Our findings support previous research that adult height is influenced strongly by the family living conditions during infancy and by community effects acting during childhood and adolescence.

## 1. Introduction

The human life cycle is characterized by an extended time for growth and development, which provides great biological plasticity, allowing human beings to adjust to changing social and environmental conditions [1]. The pre-reproductive stage of the human life cycle has critical periods in which the speed of growth is very intense—the foetal stage, infancy, and adolescence—which increase susceptibility to nutritional deficiencies or excesses, illnesses, physical workload, and psychosocial stress. These factors may affect growth irreversibly and determine health and illness patterns in the adult stage [2]. Starting with Louis-René Villermé’s seminal work of 1829 [3], *Mémoire sur la Taille de L'Homme en France*—which Darwin quotes in *The Descent of Man, and Selection in Relation to Sex* (1871)—adult height is the most commonly used indicator to evaluate ecological, socio-economic, household, and emotional conditions in which individuals of a particular population or social group grew up, attracting the interest of both human biologists and economists [4]. Since the mid-18th century, the armies of European, American, and Asian countries started measuring the height of their conscripts, thus providing the main source of data for the study of historical and social anthropometry [5], which allows for the association of trends and disparities in height to historical political events and socio-economic change [6,7,8]. Even considering the limitations of this source of data [9], the information provided by the annual time series of national measurements of conscripts offers the most solid evidence of secular trends in height as an indicator of the “biological standards of living” [10]. 

Between the middle of the 19th and the middle of the 20th centuries, there was an increase in male adult height of more than 1 cm per decade in countries undergoing industrialisation [11]. This sustained but slow increase in height in industrialized countries (as well as changes in other anthropometric and physiological variables, such as a decrease in the age of menarche [12]) is associated with the generally slow pace of global socio-economic transformation and increase of household resource allocation. Secular trends and population differences in adult height are established by the material and emotional living conditions during infancy [13,14,15] and are amplified by the influence of living conditions and social network community factors during later childhood and adolescence [14,16,17]. These changes in families and society likely operated across successive generations through epigenetic regulators of growth and development and, as Bogin has suggested [16], gradually overcame “intergenerational inertia” to increase adult height.

As a contribution to the analysis of socio-economic determinants of inequality in health, this study focuses on secular trends and social differences in adult height of conscripts born between 1916 and 1953 in the city of Madrid, Spain, a period of deep socio-economic changes and intense socio-political turmoil, including the Spanish Civil War (1936–1939) and the dictatorship of General Franco that lasted through four decades. There is in Spain a well-developed field of research for the study of secular trends in anthropometric and physiological indicators during the past 150 years, including the evaluation of differences in adult height determined by social inequalities (for a review, see [18]), and by urban vs. rural residence, especially the so-called “urban penalty” [19]. However, there are only limited studies on differences in height determined by urban social stratification. For the city of Madrid, there are only two studies on data from conscripts born in the 19th century [20,21]. Recent analyses [22,23,24] have evaluated urban differences in height in middle-sized or small Spanish cities at the turn of the 19th to 20th centuries. Likewise, at an international level, research on differences in height and socio-economic characteristics in urban districts or neighbourhoods is limited to very few studies, restricted to the United States at the end of the 20th century [25,26]. Our study aims to increase understanding of secular trends and social disparities in height in big cities during the 20th century [27]. We test the hypothesis that urban spatial segregation and social stratification were associated with significant differences in height.

## 2. Material and Methods

Data analysed correspond to the height recorded at municipal conscripting offices in Madrid City for young men called up during the period of compulsory military service in Spain, a source that was unedited and, up to now, preserved in the *Archivo General Militar de Guadalajara* (AGMG, General Military Archive of Guadalajara, Spain) [27]. Data correspond to the information regarding all young men called up in the city at the age of 21 between 1936 and 1974 (cohorts born from 1915 to 1953). Anthropometric data is not available after the mid-1970s and Spain abolished compulsory military service in 2001. Data were included in a collection of books, organized by year and district (the so-called *Libros Filiadores*), in which the personal information (filiation information, date of birth, and, occasionally, education and occupation), the conscription details (situation, deemed fit or unfit for service, and health or family allegations presented by the conscript in order to avoid or delay the immediate recruitment), and the anthropometric measurements (height, thoracic circumference, and, as from 1955, weight) of conscripts were registered. Randomly choosing the first letter of the last name, a sample of 30–40% per year and per district of all available records was collected. Data have been recorded anonymously and in accordance with the Spanish Data Protection Law (1999).

**Figure 1:**
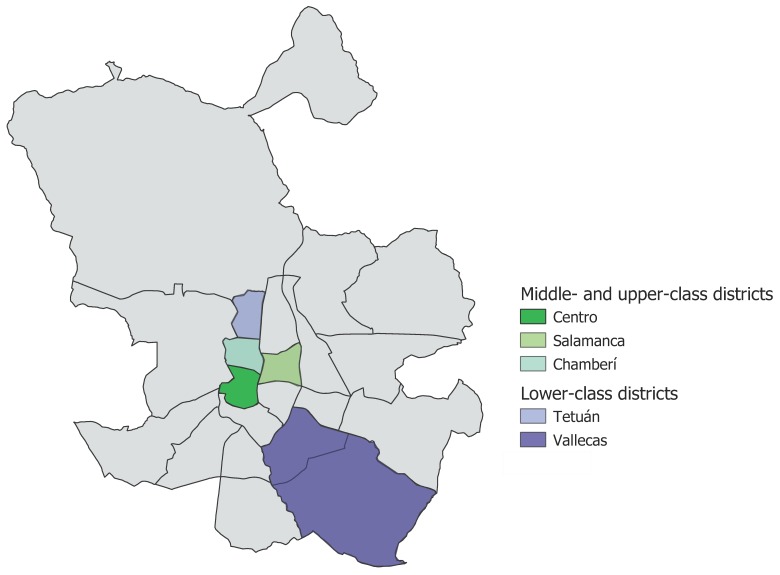
Map of Madrid and the analysed districts (modified from [34]).

Five districts of Madrid with clearly defined and contrasting socio-economic conditions have been selected and grouped in two categories [28,29]: lower class (Tetuán and Vallecas) and middle and upper classes (Centro, Salamanca and Chamberí) (Figure 1). Although the occupation and educational level of the conscripts were sometimes recorded, our analysis by districts is based on the rigid spatial segregation and social stratification of Madrid during the analysed period [30]. The two lower class districts—Tetuán and Vallecas—were slum areas respectively on the northern and south-eastern periphery of the city, characterized by improvised conurbations and unhealthy and overcrowded housing bunched around villages, which took in the flood of migrants during the first two decades of the 20th century [31,32]. On the other hand, the three middle- and upper-class districts—Centro, Salamanca, and Chamberí—included the historic centre of the city, which had been reformed and expanded into new neighbourhoods (known as the “*Ensanche*”) at the turn of the century [28,30,33].

The final sample analysed in this study corresponds to 43,633 individuals with height, which corresponded to 68.87% of the 63,355 records reviewed. The criterion was to include all individuals with height recorded, both deemed fit or unfit for military service. The majority of conscripts with no anthropometric measurements (a higher rate in the middle- and upper-class districts) were draft dodgers or volunteers who enlisted in the warring factions during the Spanish Civil War during 1936–1939 (Table 1). Of the conscripts measured, 82.52% (*n* = 36,006) were declared fit for military service, with a higher percentage considered unfit in the middle- and upper-class districts (12.96%, *n* = 2960) than the lower-class districts (5.03%, *n* = 1408).

**Figure 2:**
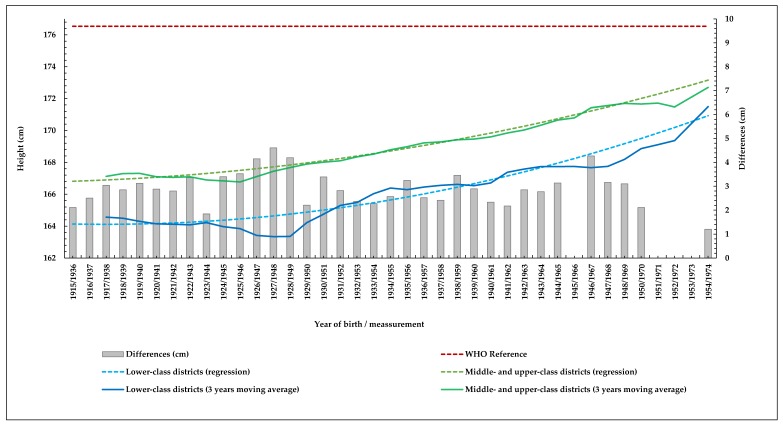
Secular trends in height in lower class districts, and middle- and upper-class districts, and annual differences between them (Source: AGMG).

**Figure 3:**
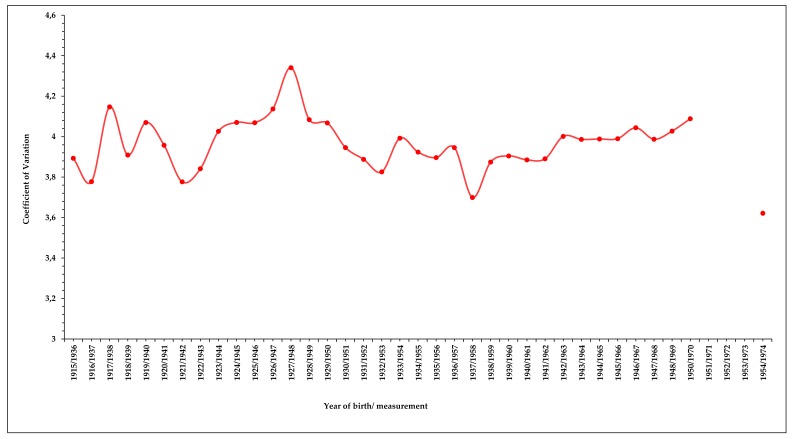
Secular trend in coefficient of variation (CV), all districts considered (Source: AGMG).

**Figure 4:**
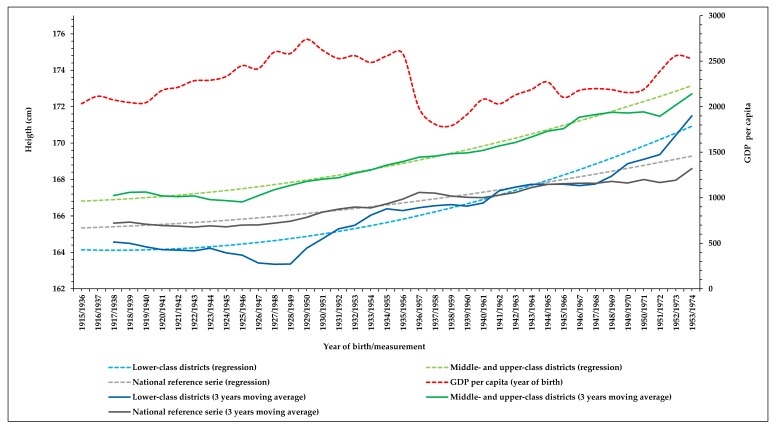
Secular trends in height in lower-class districts and middle- and upper-class districts, compared with national reference series, and trend of GDP per capita by year of birth (Sources: AGMG, [35] and [39]); *R^2^* national reference series = 0.895).

With the exception of a few lost books, the data analysed corresponded to almost every year of the period in question (Table 2). Distribution of height for the five districts and both groups are statistically normal (Kolmogorov–Smirnov test). Secular trends in height were evaluated by quadratic regression models [27]. Differences between districts for select years were evaluated by Student's *t*-test. Time series are shown in Figure 2, Figure 3 and Figure 4 as three-year moving averages in order to smooth out short-term fluctuations and highlight more long-term trends. The data for Madrid City were compared with the series by Martínez-Carrión and María-Dolores [35], established with data from conscripts living in urban and rural areas in the Mediterranean region, considered representative of the values and trends in height in Spain, given its temporal amplitude (cohorts from 1840 to 1948) and socio-economic diversity. 

Likewise, annual z-score values for both series were established, considering the value for male height at 19 years old (176.54 cm) as the average reference (percentile 50th), established by the World Health Organization’s (WHO) international growth standard [36]. 

Finally, temporal series of the coefficient of variation (CV) for all districts considered were calculated (CV = SD/men height*100, where SD is the standard deviation). The CV is the most used indicator to evaluate social inequalities expressed by differences in height [37,38]. 

Analysis were performed by SPSS (version 22, IBM Corporation, Armonk, NY, USA) and RStudio (version 3.5.1, RStudio, Inc, Boston, MA, USA) statistic programs.

## 3. Results

Height increased significantly during the analysed period in both socio-economic categories of districts, 6.75 cm in lower class districts (from 164.74 cm to 171.50, cohorts born in 1915 and 1974, respectively, *R^2^* = 0.883) and 5.85 cm in the middle and upper class ones (from 166.85 cm to 172.70 *R^2^* = 0 .951) (Table 2 and Figure 2). Correspondingly, the differences between the lower-class districts and the middle- and upper-class districts were maintained, although reduced from 2.11 cm in 1915 (*t* = 952.234; df = 1,351; *p* < 0.05) to 1.20 in 1953 (*t* = 617.429; df = 499; *p* < 0.05). Throughout the whole period, mean adult height for both categories of districts was less than the WHO international reference value, including the most recent cohorts. Correspondingly, z-scores remained between –1 and –2 standard deviations in both lower-class districts and middle- and upper-class districts (Table 2).

Despite the global positive secular trend in height of conscripts living in both categories of districts and the tendency to converge, Figure 2 shows clear differences between the lower-class districts and middle- and upper-class districts. While the positive secular trend in height among conscripts living in the middle- and upper-class districts was sustained, height among those from the lower-class districts decreased throughout the first two decades of the century (by 1.69 cm between cohorts born in 1915 and 1926), increased sharply during the first half of the 1930s, and then stabilized for more than a decade, increasing again sharply as of 1947. Because of this, the differences in height between lower-class and middle- and upper-class districts increased to a maximum value of 4.61 cm between cohorts born in 1927 (*t* = 833.347; df = 1,309; *p* < 0.05)—double that at the beginning of the series—and again to 4.27 cm between those born in 1946 (*t* = 903.398; df = 1,325; *p* < 0.05). Accordingly, urban CV increases to a maximum value of 4.3% among conscripts born in 1927, and remains around 4% throughout the decade, among those born between 1938 and 1949 (Figure 3, Table 2). 

As Figure 4 shows, throughout the analysed period, the height of conscripts who lived in middle- and upper-class districts of Madrid was noticeably higher than in the national reference series, while the height of those from lower-class districts only achieved the values of the national reference series in the cohorts of the first years of the 40s, and overtook them in the late 1940s. Figure 4 also shows the trend of GDP per capita by year of birth. The positive secular trend in height of conscripts from upper- and middle-class districts remained constant and independent of the trend in GDP per capita, while the trend in height among those from lower class districts reflects both the uneven social distribution of the benefits of the economic growth during the first decades of the century as well as the negative impact of the economic collapse from the mid-1930s and during the 1940s.

## 4. Discussion

In comparison with other capitals and big cities in other industrialized countries, Madrid delayed its definitive economic and demographic expansion until the first third of the 20th century. The demographic expansion was the result of two processes. The first one was the reduction in the very high mortality rates (especially infant mortality) as a result of the general improvements in urban sanitation (clean water supply and sewage treatment) and health conditions, which, for the first time, allowed the city a positive natural population growth [40]. At the beginning of the century, Madrid was still known as “the City of Death” because of its high mortality rates, higher than the rest of the province and the national average, and also higher than most European capitals [41].

The second determinant of the demographic growth was the massive migrant contribution, which converged on Madrid, from the whole country, especially during the 1920s after the end of the First World War. The migration was driven largely by industrial development and the rise of the construction and service sectors in the city, which required cheap, unskilled labour, as well as professionals and skilled workers [42]. Thus, during the first third of the 20th century, the metropolitan area of Madrid doubled its population, reaching over 950,000 inhabitants, while the nearby villages and outlying poor neighbourhoods—which did not become urban districts until the administrative reform of 1955—increased their population almost fivefold, reaching over 200,000 inhabitants [43]. The migrants to the more central districts of the metropolitan area were basically professionals and qualified personnel, while the outskirts attracted poor rural immigrants. This differential drawing-in of migrants to the capital determined that Madrid registered a distinct spatial segregation linked to social stratification, a characteristic shared by other expanding cities around the world, expressed by big contrasts in population density, quality of work and salary, housing cost, and sanitation and access to services between neighbourhoods and districts [30].

An extensive municipal report from 1929 [44] described these outlying slums—specifically those analysed here, Tetuán and Vallecas—as conurbations, which kept on growing “like tentacles […] with no urban planning for construction nor regulations to control them; […] with no public or private services; narrow unpaved streets, no running water, and no sewers, very often using cesspits”. Tetuán, to the north of the metropolitan area, and Vallecas, to the south-east, doubled their populations between 1920 and 1930, reaching over 39,000 and 50,000 inhabitants, respectively, more than many provincial capitals in Spain [33].

In the twenty years following the great urban transformation of Madrid at the turn of the century, the differences in epidemiological conditions and morbidity and mortality rates between districts of the city described at the beginning of the century [45,46], became established between the modernised and extended metropolitan area and its outlying suburbs in expansion [31,32]. This spatial segregation and social stratification, and the disparities in living conditions and in household income, can be associated with differences in trends in height by district during the first decades of the 20th century. The association between trends in height and infant and general mortalities is well established in such a way that, even if food were not a limiting factor, a reduction in the burden of infections would have allowed an increase in height and weight [47]. Differences in height between lower-class districts and middle- and upper-class districts were evident at the beginning of the 20th century (over 2 cm), but cohorts of conscripts of both social categories shared a stabilization or even a decline of their height in a period of economic growth, as shown in Figure 4. As disparities in living conditions—such as sanitation and overcrowding—increased, trends in height diverged despite the positive change of national macroeconomic variables [11].

The positive secular trend in height among conscripts living in the middle- and upper-class districts of Centro, Salamanca, and Chamberí started in the mid-1920s and remained stable during the following decades, while height of conscripts from the lower-class areas of Tetuán and Vallecas reduced by 1.69 cm among the cohorts born between 1915 and 1926. Height of conscripts growing in the slum areas increased sharply only during the next decade, a period of economic stabilization characterised by improvements in sanitation and socio-economic conditions [39], particularly after the overthrow of the Spanish monarchy and the proclamation of the republic following the elections of April 1931, a period of high expectations and advances for the working and popular classes. However, while adult height continued to increase among the conscripts of middle and upper classes in the following decades, height of those from the lower-class did not increase, or increased very slowly, from the mid-1930s until the last years of the 1940s. This was the extended period of economic breakdown (Figure 4), first determined by the Spanish Civil War (1936–1939) and later by the dictatorship of General Franco. These events induced a decade of mass repression and social setbacks, acute economic crisis, and international isolation of the so-called “autarchy period” [48]. Height among conscripts born in lower-class districts only increase clearly again in cohorts born in the first years of the 1950s. In stark contrast, the cohorts born in middle- and upper-class districts during these two decades of civil conflict and economic collapse maintained a steady increase in adult height, with a final height over 172 cm (cohort born in 1953). Differences in height remained at the end of the period (1.20 cm, cohort born in 1953), in spite of height of conscripts born in the lower-class district increasing faster than among their peers of middle and upper-class districts. Correspondingly, the CV increased to over 4% in mid-1920s and remained around this value during the next two decades, over those values established for European [49] and Spanish military series [50,51].

The nature of conscription data does not allow determining in which pre-adult period of the life cycle the differences in height among groups of the same cohort who experience different socio-economic conditions during their growth were established. Longitudinal studies [13,52,53] have shown that both secular trends, as well as differences in adult height in human populations, are established very early, at about two years of age, although there is also evidence of the influence of later childhood and adolescence on final adult height [16,54]. Our results seem to conform well to the existing literature. In the lower-class districts, the period of most intense reduction in height stretches from the early 1920s to the beginning of the 1930s, the period of uncontrolled demographic expansion in these settlements on the outskirts of Madrid. It was in this period that the first cohorts of conscripts included in our analysis were born or spent their infancy, sons of the poorest immigrants arriving to Madrid as young singles of either sex or as young couples [55]. The offspring of these immigrants faced the negative impact of the dismal living conditions during their infancy. As children and juveniles, they also suffered a very early incorporation to the labour force [56] and, finally, in their adolescence, an extended period of hardships and famine during the Civil War and first decade of the regime of General Franco. Likewise, accumulative negative impacts on growth can be expected in those born from the mid-1930s onwards. In sum, as growth in height is an accumulative process, conditions influencing growth need to be understood cumulatively [57], from a panoramic life cycle perspective [4].

Additionally, factors other than nutrition, living conditions, and health might also play a relevant role in regulating growth. These factors are gathered under the designation of “community-based factors on growth” or “community effects” [58] and refer to social connectedness and expectations affecting growth in a population as a whole, highlighting the impact of the social-emotional environment on child and adolescent growth. This new field of research is based on Social Identity Theory and some of our results fit well with the predictions of this theory [59]. Social identity theory considers that a portion of an individual's concept of self is derived from perceived membership in a relevant social group in such a way that peer group membership correlates with emotional, evaluative, and other psychological parameters, as well as behaviours and, finally, biological variables [60,61]. 

The community effect hypothesis predicts that short stature communities generate short people, as is the case of the urban population analysed here. In accordance with this theory, the adult height of conscripts living in both the lower-class districts and the middle- and upper-class districts was well below the international reference value established by WHO for age 19 years, 176.54 cm [36]. The z-score of those born in lower-class districts in the 1920s is close to a value of –2 standard deviations, which, in children, is considered the cut-off point for impaired growth and development (growth stunting). But likewise, the height of conscripts in middle- and upper-class districts also shows z-scores with values at minus one standard deviation below the reference value for almost the entire analysed period. The rate of increase of male height was generally slower in Spain than in other European countries, but more intense in the cohorts born since the 1950s [11]. Adult male height in Spain reached the WHO reference average of 176.54 cm finally in the cohort born in 1996 [62]. In other words, the conscripts from middle- and upper-class districts who grew up in relatively good material conditions maintained adult heights smaller than expected. Biological mechanisms underlying the community effect are beginning to be described [63] and relate to the ways in which social class conflict and emotional stress are transduced by the neuroendocrine system into hormones that restrict skeletal growth. 

The extended period of intense political instability and social conflict, of deep-seated social inequalities, violence, and repression that characterized Spain, at least during the first half of the 20th century, affected growth, beyond the lower status working-class and the even more disadvantaged classes, to the wealthier sector of society. Analysis of modern recent conflicts have shown similar negative consequence for height growth [16]. Another prediction of the community effects hypothesis is that the secular trend is a shift in *toto*, such that the variation in height does not change when the average population height increases [17]. Our analysis found that this is the case for the Madrid urban population of contrasting socio-economic categories. 

## 5. Conclusions

This contribution to the evaluation of the impact of social and family determinants on growth adjustment aims to provide a perspective of population analysis for a large European city during the first half of the 20th century. Results confirm differences in height that can be associated with the spatial segregation and social stratification that were characteristic of the city of Madrid throughout the period analysed. Large cities house notorious inequalities that negatively affect the biological wellbeing of their least privileged dwellers throughout their life cycle, critically, during the most sensitive stages of growth and development, as recent studies on neighbourhood disadvantage and depressive symptoms among adolescents have made clear [64]. Our analysis implicates the effects of biological living conditions and community effects acting during infancy, childhood and adolescence on social class differences in height.

## Figures and Tables

**Table 1:** Distribution of data of conscripts by district and availability of anthropometric measurements (Source: AGMG).

Districts	With Measurements	Without Measurements				
		Voluntary	Excluded	Auxiliary Services	Draft Dodges	Others
	**% (*n*)**					
**Lower Class**	76.81 (20,809)	14.46 (3918)	0.79 (214)	0.06 (17)	4.27 (1158)	3.60 (974)
**Middle and Upper Classes**	62.94 (22,824)	23.53 (8532)	0.81 (295)	0.05 (19)	7.42 (2692)	5.25 (1903)
**Total**	68.87 (43,633)	31.13 (19,722)				

**Table 2:** Mean height and z-score by year in lower, middle, and upper class districts, and total mean height, z-score, and coefficient of variation (CV) (Source: AGMG).

Year of Birth/Measurement	Lower Class Districts			Middle and Upper class Districts			Mean Height of All Districts			
	N	Mean (SD)	Z Score	N	Mean (SD)	Z Score	N	Mean (SD)	Z Score	CV
**1915/1936**	435	164.74 (6.25)	−1.73	650	166.85 (6.47)	−1.42	1085	166.00 (6.46)	−1.55	3.9
**1916/1937**	382	164.60 (5.79)	−1.75	303	167.10 (6.55)	−1.39	685	165.70 (6.26)	−1.59	3.8
**1917/1938**	392	164.37 (6.71)	−1.79	409	167.41 (6.72)	−1.34	801	165.92 (6.88)	−1.56	4.1
**1918/1939**	380	164.52 (6.01)	−1.77	401	167.37 (6.62)	−1.35	781	165.98 (6.49)	−1.55	3.9
**1919/1940**	536	164.02 (6.08)	−1.84	587	167.14 (6.97)	−1.38	1123	165.65 (6.74)	−1.60	4.1
**1920/1941**	664	163.90 (6.28)	−1.86	421	166.78 (6.54)	−1.43	1085	165.02 (6.53)	−1.69	4.0
**1921/1942**	498	164.45 (6.12)	−1.78	815	167.25 (6.13)	−1.36	1313	166.19 (6.28)	−1.52	3.8
**1922/1943**	567	163.89 (6.07)	−1.86	795	167.25 (6.21)	−1.36	1362	165.85 (6.37)	−1.57	3.8
**1923/1944**	491	164.33 (6.33)	−1.79	796	166.17 (6.77)	−1.52	1287	165.47 (6.66)	−1.63	4.0
**1924/1945**	563	163.69 (6.04)	−1.89	813	167.09 (6.86)	−1.39	1376	165.7 (6.74)	−1.59	4.1
**1925/1946**	476	163.52 (6.56)	−1.91	793	167.04 (6.51)	−1.40	1269	165.72 (6.74)	−1.59	4.1
**1926/1947**	549	163.05 (6.33)	−1.98	696	167.19 (6.68)	−1.37	1245	165.36 (6.84)	−1.64	4.1
**1927/1948**	500	163.47 (6.80)	−1.92	653	168.08 (6.87)	−1.24	1153	166.08 (7.21)	−1.54	4.3
**1928/1949**	914	163.55 (6.24)	−1.91	1141	167.75 (6.61)	−1.29	2055	165.88 (6.77)	−1.57	4.1
**1929/1950**	510	165.64 (6.66)	−1.60	459	168.44 (6.91)	−1.19	1313	166.23 (6.56)	−1.51	3.9
**1930/1951**	854	165.05 (6.18)	−1.69	695	168.43 (6.91)	−1.19	1313	166.23 (6.56)	−1.51	4.0
**1931/1952**	517	165.19 (6.23)	−1.67	487	168.01 (6.42)	−1.25	1004	166.56 (6.48)	−1.47	3.9
**1932/1953**	554	166.21 (6.28)	−1.52	477	168.58 (6.32)	−1.17	1031	167.31 (6.40)	−1.36	3.8
**1933/1954**	248	166.69 (6.72)	−1.45	749	168.97 (6.63)	−1.11	994	168.4( 6.72)	−1.28	4.0
**1934/1955**	813	166.26 (6.30)	−1.51	717	168.82 (6.60)	−1.13	1530	167.46 (6.57)	−1.33	3.9
**1935/1956**	616	165.91 (6.08)	−1.56	766	169.16 (6.53)	−1.08	1382	167.71 (6.53)	−1.30	3.9
**1936/1957**	918	167.17 (6.07)	−1.38	712	169.69 (7.07)	−1.01	1630	168.27 (6.64)	−1.22	3.9
**1937/1958**	640	166.59 (5.75)	−1.46	702	169.00 (6.39)	−1.11	1342	167.85 (6.21)	−1.28	3.7
**1938/1959**	790	166.09 (6.10)	−1.53	1020	169.55 (6.42)	−1.03	1810	168.04 (6.51)	−1.25	3.9
**1939/1960**	646	166.92 (6.32)	−1.41	761	169.82 (6.51)	−0.99	1407	168.49 (6.58)	−1.18	3.9
**1940/1961**	782	167.10 (6.29)	−1.39	713	169.43 (6.58)	−1.04	1495	168.21 (6.54)	−1.22	3.9
**1941/1962**	260	168.11 (5.93)	−1.24	703	170.29 (6.74)	−0.92	963	169.7 (6.60)	−1.01	3.9
**1942/1963**	784	167.53 (6.45)	−1.32	743	170.38 (6.77)	−0.90	1527	168.92 (6.76)	−1.12	4.0
**1943/1964**	628	167.55 (6.08)	−1.32	774	170.33 (6.99)	−0.91	1402	169.08 (6.74)	−1.10	4.0
**1944/1965**	877	168.12 (6.49)	−1.24	726	171.25 (6.69)	−0.78	1603	169.54 (6.76)	−1.03	4.0
**1945/1966**	627	167.55 (6.69)	−1.32	−−−	−−−	−−−	627	167.55 (6.69)	−1.32	4.0
**1946/1967**	621	167.32 (6.75)	−1.35	500	171.59 (6.19)	−0.73	1121	169.23 (6.84)	−1.07	4.0
**1947/1968**	637	168.38 (6.79)	−1.20	488	171.54 (6.31)	−0.74	1125	169.75 (6.77)	−1.00	4.0
**1948/1969**	648	168.85 (6.54)	−1.13	522	171.95 (6.86)	−0.67	1170	170.24 (6.86)	−0.93	4.0
**1949/1970**	200	169.37 (6.86)	−1.05	200	171.47 (6.93)	−0.74	400	170.42 (6.97)	−0.90	4.1
**1953/1974**	300	171.50 (6.15)	−0.74	200	172.70 (6.29)	−0.56	500	171.98 (6.23)	−0.67	3.6
